# Adverse childhood experiences among adults with eating disorders: comparison to a nationally representative sample and identification of trauma

**DOI:** 10.1186/s40337-022-00594-x

**Published:** 2022-05-20

**Authors:** Renee D. Rienecke, Craig Johnson, Daniel Le Grange, Jamie Manwaring, Philip S. Mehler, Alan Duffy, Susan McClanahan, Dan V. Blalock

**Affiliations:** 1 Eating Recovery Center and Pathlight Mood & Anxiety Centers, Denver, USA; 2 Department of Psychiatry and Behavioral Sciences, Northwestern University, 333 N. Michigan Avenue, Ste. 1900, Denver, IL 60601 USA; 3 Department of Psychiatry and Behavioral Sciences, University of California, San Francisco, CA USA; 4 Department of Psychiatry and Behavioral Neuroscience, The University of Chicago, Chicago, IL (Emeritus) USA; 5 ACUTE, at Denver Health, Denver, CO USA; 6 Department of Medicine, University of Colorado, Denver, CO USA; 7 Rush University Medical Center, Denver, IL USA; 8 Center of Innovation to Accelerate Discovery and Practice Transformation, Durham Veterans Affairs Medical Center, Durham, NC USA; 9 Department of Psychiatry and Behavioral Sciences, Duke University School of Medicine, Durham, NC USA

**Keywords:** Adverse childhood experiences, Eating disorders, Latent class analysis, Adults

## Abstract

**Background:**

Adverse childhood experiences (ACEs) are prevalent, impact long-term physical and mental health, and are associated with eating disorders (EDs) in adulthood. The primary objectives of the current study were: (1) to examine and compare ACEs between two samples: treatment-seeking adults, and a nationally representative sample of adults, (2) to characterize ACEs items and total scores across demographic and diagnostic information in adults seeking treatment for an ED, (3) to statistically classify ACEs profiles using latent class analysis, and (4) to examine associations between ACEs profiles and diagnosis.

**Methods:**

This cross-sectional study assessed patients with a DSM-5 ED receiving treatment between October 2018 and April 2020 at the inpatient, residential, or partial hospitalization levels of care at one of two private ED treatment facilities. ACEs were assessed with the Adverse Childhood Experiences Survey at admission. Generalized linear models and Welch’s t-tests were used to compare ACEs in the current sample with national estimates. A latent class analysis was conducted to examine subgroups of ACEs responses, and differences in these classes by ED diagnoses were examined with multinomial logistic regression.

**Results:**

Patients with EDs had significantly higher ACEs scores (*M* = 1.95, *SD* = 1.90) than the nationally representative sample (*M* = 1.57, *SD* = 4.72; *t* = 6.42, *p* < .001). Within patients with EDs, four latent classes of ACEs item endorsement were identified. Patients with other specified feeding or eating disorder (OSFED) and binge eating disorder (BED) were more likely to fall into the “Household ACEs” and “Abuse ACEs” groups, respectively, compared to anorexia nervosa—restricting subtype (AN-R).

**Conclusions:**

Patients with EDs reported more ACEs than the nationally representative sample, and differences in total ACEs and latent class membership were found across ED diagnoses. The current study can inform the development of trauma-informed care for patients with EDs.

**Supplementary Information:**

The online version contains supplementary material available at 10.1186/s40337-022-00594-x.

## Plain English summary

Adverse childhood experiences (ACEs), such as abuse, are considered a significant public health crisis and are associated with mental and physical health problems later in life. The current study compared ACEs among a treatment-seeking sample of adults with eating disorders to a nationally representative sample, and found that patients with eating disorders reported higher ACEs scores. Within the eating disorder sample, females were more likely to report a history of sexual abuse than males. Four categories of ACEs emerged, representing four distinct clusters of ACEs item endorsement. Patients with binge eating disorder (BED) reported higher levels of ACEs than patients with anorexia nervosa – restricting subtype (AN-R). In addition, patients with other specified feeding or eating disorder (OSFED) were more likely to be characterized by high levels of household dysfunction than patients with AN-R. Screening for adverse childhood experiences among patients with eating disorders should be part of standard care, and more broadly, providing children with safe environments may lessen the long-term development of several serious illnesses, including eating disorders.

**Supplementary Information:**

The online version contains supplementary material available at 10.1186/s40337-022-00594-x.

Exposure to adverse experiences in childhood can set the stage for long-term negative health outcomes and health disparities across the life span [1], and as such have been referred to as a significant public health burden “that could rival or exceed all other root causes” [2]. Adverse childhood experiences (ACEs) include emotional, physical, or sexual abuse, neglect, or family experiences such as incarceration of a household member. A nationally representative sample of 214,157 adults found that almost a quarter (23.5%) reported one ACE and 15.8% reported four or more ACEs [1]. ACEs are associated with long-term adverse impacts; they have been shown to predict numerous poor mental and physical health outcomes in adulthood, including suicide attempts, drug abuse, obesity, heart disease, and decreased life expectancy [3–5]. The strong link between traumatic and unsafe experiences in childhood, and physical and mental health in adulthood, has led to a policy statement being released by the American Academy of Pediatrics stating that reducing toxic stress in childhood should be a leading priority for the field of medicine [6].

In addition to being highly prevalent among patients with psychiatric disorders such as anxiety or depression [7], childhood maltreatment is associated with eating disorders (ED) and ED behaviors in adulthood [8–11]. EDs are prevalent [12] and serious disorders associated with significant comorbidity [13], high rates of mortality [14], and impaired quality of life [15]. Among clinical samples, a meta-analysis found that EDs are associated with childhood sexual, physical, and emotional abuse [16]. Individuals with EDs also report higher rates of childhood maltreatment than healthy controls or psychiatric control groups [17]. Patients with bulimia nervosa (BN) report more moderate to severe trauma histories than those with anorexia nervosa (AN) [18].

Although several studies of EDs and trauma have focused on childhood sexual abuse, a more detailed view of trauma histories would assess multiple types of trauma, as a significant minority of youth (40.9%) report more than one direct exposure to violence, abuse, or crime [19]. One way to examine complex histories of abuse is to use methods such as latent class analysis (LCA) to identify latent clusters of adverse events that may be more likely to occur together. As opposed to variable-centered analyses that examine associations among variables across an entire sample with little consideration of person-level prediction, person-centered analyses such as LCA allow observation of and prediction based on complex associations within each individual. In these analyses, a real person becomes the unit of analysis rather than a more abstract variable. As such, these results are more immediately translatable to person-specific inferences and predictions.

Given the prevalence of adverse childhood experiences, their association with EDs, and the impairment with which they are associated, trauma-informed care (TIC) has been increasingly recommended as part of the treatment approach for patients with both EDs and a history of trauma or comorbid posttraumatic stress disorder [20]. To properly design and implement TIC for the broader ED patient population, as well as specific symptomatology within ED patients and ED diagnoses, it is important to know the prevalence and severity of their adverse childhood experiences, and to know whether some individuals may have a more significant childhood history of trauma than others.

The primary aims of the current study were:

1. to examine and compare ACEs between two samples: adults seeking treatment for an ED, and a nationally representative sample of adults [1], and
2. to characterize ACEs items and total scores across demographic information in adults seeking treatment for an ED.

It was hypothesized that the ED group would report a greater number of ACEs than the nationally representative sample.

Secondary aims included:

- 3) to statistically classify ACEs profiles using LCA, and.
- 4) to examine associations between ACEs profiles and diagnosis.

No a priori hypotheses were made regarding the ACEs profiles. This is the first study to compare ACEs in a large sample of patients with EDs to a nationally representative sample of adults.

## Methods

### Participants and procedure

Participants were 1061 adult patients with an ED as determined by *Diagnostic and Statistical Manual of Mental Disorders—5th edition* (DSM-5) [21] criteria receiving treatment at two Eating Recovery Center (ERC) facilities in the U.S. ERC is a private treatment facility offering higher levels of care for EDs. Diagnoses were made at each patient’s intake appointment, prior to beginning treatment, by a licensed clinician. Semi-structured interviews were conducted to assess ED symptoms. Participants were treated between October 1, 2018 and April 30, 2020 at the inpatient, residential, partial hospitalization, or intensive outpatient levels of care at one of the two facilities. Patients provided informed consent at baseline and completed self-report measures at admission. The nationally representative sample consisted of 214,157 adults [1]. This study was approved by Salus Institutional Review Board.

### Measures

The *Adverse Childhood Experiences Survey (ACES)* [3] is a 10-item self-report measure assessing childhood trauma, including physical, emotional, or sexual abuse, emotional or physical neglect, having a mother or stepmother who is a victim of intimate partner violence, having divorced parents, having a family member diagnosed with a mental illness or who attempted suicide, having a family member in prison, and having a parent who struggles with alcoholism or drug use. The ACES has been used in numerous populations and has been shown to be a strong predictor of physical and mental health disorders later in life [2]. For comparison purposes, the 8 ACEs items consistent with Merrick et al. [1] were used in the current study (not including emotional or physical neglect). Each affirmative response is given one point; the points are then added for a total score up to 8 points.

### Statistical analyses

All analyses were performed in R (version 4.1.1) [22]. Generalized linear models with odds ratios and 95% confidence intervals were used to examine differences within the ED sample by diagnosis, gender identity, and race. Welch’s t-test was used to compare the ED sample ACES with the nationally representative sample to account for unequal variances. Because the ED sample was overwhelmingly comprised of female patients and white patients, supplemental comparisons were performed on these patients with only female or white participants in the nationally representative sample to investigate any sample differences that may have arisen due to differential demographic makeup between samples. Importantly, additional comparisons between other genders and other races could not be performed due to low cell sizes in the ED sample. Multinomial logistic regression models were used to examine associations of demographic variables and treatment arm with class membership.

An iterative LCA method was used (with the R statistical package poLCA) [23] to determine the number of classes that underlie endorsement of the eight ACEs items. For each model, LCA uses a maximum likelihood estimation algorithm to obtain the probabilities of an individual falling within each class, as well as the conditional probability of endorsing each specific ACE given membership in a specific class. The result is individual patients separated into a discrete number of classes based on their chances of endorsing a specific pattern across all eight ACEs items. In short, we grouped patients according to their patterns of ACEs endorsement.

We used the log likelihood function, Akaike's Information Criterion (AIC), and Bayesian Information Criterion (BIC) to assess fit between the latent class model results and the data. In addition, we used Entropy to examine the separation of classes within each solution, where higher Entropy indicates better separation of classes. Discrepant results among the indicators led us to favor the BIC fit statistic to account for parsimony of the model; our sample size was also not underpowered, which would have led us to favor log likelihood or AIC [24]. To avoid the problems of local maxima in the maximum likelihood estimation algorithm, five hundred iterations were performed on each of the models. Each model converged in less than one hundred iterations, suggesting little difficulty with local maxima.

A multinomial logistic regression was used to predict categorical latent class assignment from diagnosis, with the “Low ACEs” latent class representing the reference outcome model, and a diagnosis of anorexia nervosa—restricting subtype (AN-R) representing the reference group of the diagnosis predictor. Both the “Low ACEs” latent class and patients with a diagnosis of AN-R were chosen as references because they represent a clear “de-facto” group to which to compare, as patients with AN-R have repeatedly been found to report lower rates of childhood abuse than patients with other ED diagnoses [9, 25]. Both one-step and three-step models were considered for performing the multinomial logistic regression on our latent class solution [26]. As entropy was found to be sufficiently high, and thus with the class probabilities for each individual sufficiently high (or low), we chose to perform the multinomial logistic regression on the predicted latent class scores.

## Results

Participants (N = 1061) with EDs were primarily female (88.2%) and white (83%), representative of most ED patients [27]. Most patients (71.5%) reported at least one ACE. See Table 1 for patient demographic characteristics.

**Table 1:** ED patient characteristics

Age (M, SD)					27.14 (10.15) Range: 17–72				
ACES (M, SD)					1.95 (1.90)				
ACES Score	0	1	2	3	4	5	6	7	8
N	302	231	175	142	87	61	36	19	8
*Diagnosis (N, %)*									
AN-R					278 (26.2%)				
AN-BP					298 (28.1%)				
BN					134 (12.6%)				
ARFID					146 (13.8%)				
BED					78 (7.4%)				
OSFED					127 (12.0%)				
*Gender (N, %)*									
Female					936 (88.2%)				
Male					114 (10.7%)				
Nonbinary					6 (0.6%)				
Trans Female-to-Male					3 (0.3%)				
Trans Male-to-Female					0 (0.0%)				
Prefer Not to Answer					1 (0.1%)				
*Race (N, %)*									
White					881 (83.0%)				
Black					4 (0.4%)				
Hispanic					40 (3.8%)				
Asian					28 (2.6%)				
Multiracial					42 (4.0%)				
Native American/Pacific Islander					5 (0.5%)				
Declined/Other					61 (5.7%)				

*AN-R* anorexia nervosa—restricting type; *AN-BP* anorexia nervosa – binge/purge type; *BN* bulimia nervosa; *ARFID* avoidant/restrictive food intake disorder; *BED* binge eating disorder; *OSFED* other specified feeding or eating disorder

### Patients with EDs compared to nationally representative sample

Patients in the nationally representative sample were primarily white (68.08%), employed (54.15%), attended some college (32.56%), had a household income over $50,000 (43.54%), and identified as heterosexual (96.02%). Information on exact age range and presence of comorbid psychiatric illnesses was not available [1].

Patients with an ED diagnosis in the current sample (*M* = 1.95, *SD* = 1.90) had significantly higher mean ACEs total scores than the nationally representative sample (*M* = 1.57, *SD* = 4.72; *t* = 6.42, *p* < 0.001). Patients within each ED diagnosis had significantly higher mean ACEs scores than the nationally representative sample (see Table 2). Patients with EDs were significantly more likely than the nationally representative sample to endorse sexual abuse, household divorce, and household mental illness. Patients with EDs were significantly less likely to endorse household intimate partner violence and a household member having been in prison (see Table 3).

**Table 2:** ED sample compared to nationally representative sample by ED Diagnosis

Sample (N)	Sample mean ACEs (SD)	Nat’l mean ACEs (SD)	t-value	*p*-value	cohen’s d
AN-R (278)	**1.82 (1.93)**	**1.57 (4.72)**	**2.15**	**.03**	**.05**
AN-BP (298)	**1.89 (1.87)**	**1.57 (4.72)**	**2.94**	** < .01**	**.07**
BN (134)	**1.99 (1.86)**	**1.57 (4.72)**	**2.61**	**.01**	**.09**
ARFID (146)	**1.93 (1.86)**	**1.57 (4.72)**	**2.33**	**.02**	**.08**
BED (78)	**2.42 (2.04)**	**1.57 (4.72)**	**3.68**	** < .001**	**.18**
OSFED (127)	**2.12 (1.86)**	**1.57 (4.72)**	**3.33**	**.001**	**.12**

Bolded numbers represent significant differences. “Nat’l” refers to the nationally representative sample described in Merrick et al., 2018 [1], N = 214,157

**Table 3:** ED sample compared to nationally representative sample

Total Sample									
Sample	**ACEs M (SD)**	Emo Abuse (%)	Phys Abuse (%)	**Sex Abuse **(%)	**Divorce **(%)	**IPV **(%)	Sub Use (%)	**Mental Illness **(%)	**Prison **(%)
ED	**1.95 (1.90)**	35	16	**19**	**35**	**9**	29	**42**	**6**
Nat’l	**1.57 (4.72)**	34	17	**11**	**27**	**17**	27	**16**	**7**

Endorsement in ED total sample compared to nationally representative total sample									
Test	t-test, *p*-value	OR, 95% CI	OR, 95% CI	OR, 95% CI	OR, 95% CI	OR, 95% CI	OR, 95% CI	OR, 95% CI	OR, 95% CI
Result	**t = 6.42, *p* < .000000001**	1.05 [0.93, 1.19]	0.93, [0.79, 1.09]	**1.87, [1.61, 2.18]**	**1.46, [1.29, 1.66]**	**0.48, [0.39, 0.60]**	1.11, [0.98, 1.27]	**3.70, [3.28, 4.19]**	**0.75, [0.58, 0.96]**
	**cohen’s d = .08**								

Bolded numbers represent significant differences. “ED” refers to sample of patients seeking treatment for ED. “Nat’l” refers to the nationally representative sample described in Merrick et al., 2018.^1^ “Divorce” refers to household divorce. “IPV” refers to a mother or stepmother in the household experiencing intimate partner violence. “Sub Use” refers to having a family member in the household engaging in illicit drug use or struggling with alcoholism. “Mental Illness” refers to having a family member in the household who struggles with mental illness or has attempted suicide. “Prison” refers to having a family member in the household who is incarcerated

When comparing patients with EDs to the nationally representative sample subset by gender (female only) and race (white only) to account for the disproportionate demographic features in patients with EDs, mean ACEs scores in ED patients remained significantly higher, and differences in individual ACE endorsement between the samples were all retained except that endorsement of a household member having been to prison was no longer significantly different (see Additional file 1: Tables S1 and S2).

### Demographic differences in ACEs scores within the ED group

Due to low sample sizes for gender identity categories other than male/female (each < 1%), statistical comparisons could only be made for male/female. No significant gender differences existed for total ACEs score. Males were less likely to report a history of sexual abuse than females (11% versus 20%; OR = 2.18, 95% C.I. [1.22, 4.25]). Due to low sample sizes for other racial/ethnic categories, only statistical comparisons among white, Hispanic, and Asian patients could be made. Only Asian patients (*M* = 1.21, *SD* = 1.45) had significantly lower ACEs scores than white patients (*M* = 1.96, *SD* = 1.93; *t* = − 2.05, *p* = 0.04). Hispanic patients were more likely to report a history of sexual abuse than white patients (32% versus 20%; OR = 1.98, 95% C.I. [1.002, 3.93]), and were more likely to report household incarceration than white patients (22% vs. 6%; OR = 4.83, 95% C.I. [2.18, 10.70]). Asian patients were less likely to report a history of household mental illness than white patients (21% vs. 42%; OR = 0.37, 95% C.I. [0.15, 0.93]).

Only patients with a binge eating disorder (BED) diagnosis had significantly higher ACEs total scores than AN-R (*M* = 2.42, *SD* = 2.04 versus *M* = 1.82, *SD* = 1.93, respectively). Patients with BED (31%) were significantly more likely than patients with AN-R (17%) to endorse physical abuse (b = 0.81, *p* < 0.01). Patients with other specified feeding or eating disorder (OSFED) were significantly more likely than patients with AN-R to endorse emotional abuse (44% for OSFED versus 31% for AN-R; b = 0.58, *p* < 0.01) and household substance use (36% for OSFED versus 25% for AN-R; b = 0.52, *p* = 0.02). Patients with BN (35%) were significantly more likely than patients with AN-R (25%) to endorse household substance use (b = 0.47, *p* = 0.04). Patients with anorexia nervosa—binge/purge subtype (AN-BP) and avoidant/restrictive food intake disorder (ARFID) were not significantly more likely than patients with AN-R to endorse any item (all *p*’s > 0.13).

### Latent class analysis

Based on model fit indices, entropy values, and theoretical examination of the latent classes of each model, the 4-class model was chosen as the optimal model solution, though models were tested up to a 7-class solution (see Additional file 1: Table S3).

The largest subgroup (47.4%) was labeled as the “Low ACEs” subgroup, as all probabilities of ACE endorsement fell below 17%. The second largest group (32.7%) was labeled as the “Household ACEs” subgroup, as the only ACEs with a probability of endorsement above 50% were household divorce, household substance use, and household mental illness. The third largest group (10.6%) was labeled as the “All ACEs” subgroup, as all ACEs had a probability of endorsement above 60%, except household incarceration (which was still above 25% and over three times more likely than any other subgroup). The smallest subgroup (9.5%) was labeled as the “Abuse ACEs” subgroup, as the only ACEs with a probability of endorsement above 50% were physical abuse, emotional abuse, and household mental illness (which had a lower probability of endorsement than the “Household ACEs” subgroup) (see Table 4).

**Table 4:** ACE item endorsement probability by latent class

	“All ACEs” subgroup (%)	“Abuse ACEs” subgroup (%)	“Household ACEs” subgroup (%)	“Low ACEs” subgroup (%)
Emotional Abuse	***100***	**86.76**	37.21	09.89
Physical Abuse	**66.67**	***100***	02.37	00.00
Sexual Abuse	***65.24***	34.08	20.57	06.06
Divorce	***73.38***	41.30	**52.20**	14.47
IPV	***60.09***	13.51	05.39	00.00
Substance Use	***83.54***	25.41	**50.42**	04.39
Mental Illness	***91.10***	**54.56**	**60.09**	16.15
Prison	*26.73*	02.97	07.80	00.72

Bolded probabilities are > 50%. Italicized probabilities are highest probability across subgroups, irrespective of raw %

### Associations between ACEs profiles and diagnosis

The diagnostic breakdown for each subgroup can be seen in Table 5. Multinomial logistic regression revealed two significant comparisons based on latent class and diagnosis. The odds of being in the “Household ACEs” subgroup (versus the “Low ACEs” subgroup) were significantly higher in patients with OSFED than patients with AN-R (OR = 1.65, 95% C.I. [1.03, 2.66]). The odds of being in the “Abuse ACEs” subgroup (versus the “Low ACEs” subgroup) was significantly higher in patients with BED than patients with AN-R (OR = 2.80, 95% C.I. [1.30, 6.02]).

**Table 5:** Proportions of latent subgroups by diagnosis

Class Name, N (%)	AN-R^‡^ N (%)	AN-BP N (%)	BN N (%)	ARFID N (%)	BED N (%)	OSFED N (%)
“Low ACEs” ^†^ 503 (47%)	145^†^ (52%)	147^†^ (49%)	62^†^ (46%)	68^†^ (46%)	29^†^ (37%)	52^†^ (41%)
“Household ACEs” 347 (33%)	81^‡^ (29%)	99 (33%)	47 (35%)	48 (33%)	24 (31%)	**48 (38%)**
“Abuse ACEs” 99 (9%)	25^‡^ (9%)	20 (7%)	10 (7%)	17 (12%)	**14 (18%)**	13 (10%)
“All ACEs” 112 (10%)	27^‡^ (10%)	32 (11%)	15 (11%)	13 (9%)	11 (14%)	14 (11%)

^†^Reference group of “Low ACEs” latent subgroup in categorical outcome of the multinomial logistic regression. ^‡^Reference group of patients with AN-R in the categorical predictor of the multinomial logistic regression. Bolded numbers represent a significant increase in the risk ratio of being in the identified latent subgroup row (vs. the “Low ACEs” subgroup) for patients with the identified diagnosis column (vs. patients with AN-R)—a comparison across four total prevalence rates. No inferential comparisons have been made between any two prevalence rates. Latent subgroup row percentages add up to 100% down each diagnosis column. Analysis was multinomial logistic regression

## Discussion

Adverse childhood experiences have repeatedly been shown to be associated with physical and mental health disorders later in life and represent a significant public health crisis [2]. The purpose of the current study was to explore demographic differences in ACEs scores for adults with EDs, to compare ACEs between an adult sample of patients seeking treatment for an ED and a nationally representative sample, to establish ACEs profiles, and to examine ACEs profiles and their association with ED diagnoses.

The current study is the first to compare ACEs between patients with EDs and a nationally representative sample of adults. The ED sample was found to report higher ACEs scores, as hypothesized, and these results remained consistent for each individual ED diagnosis. The ED sample also reported more sexual abuse, and further analyses comparing only females suggested that these differences were unlikely to be due to differences in the gender makeup of the samples. Parental divorce and mental illness were also higher in the ED group, also consistent with previous studies comparing patients with EDs to healthy controls [17]. Patients with EDs were less likely to endorse household intimate partner violence and a household member having been in prison. To the authors’ knowledge, this has not been reported before and findings should be replicated. What remains unclear is the mechanism by which ACEs are associated with the development of EDs. Longitudinal studies are needed to better clarify this relationship, and to identify under which circumstances an individual who has experienced an ACE develops an ED as opposed to, for example, a depressive disorder. Although information on psychiatric disorders in the nationally representative sample was not available, there is no indication that those with comorbid psychiatric disorders were excluded from the sample [1]. Indeed, as it was intended to be nationally representative, one may expect that rates of comorbid psychiatric illnesses reflect national averages.

Within the ED sample, although males reported less history of sexual abuse than females, which is consistent with previous research [28], there were no differences in overall ACEs scores between males and females, which is inconsistent with findings from previous studies [1, 29]. Racial differences in ACEs in the current paper were consistent with previous research finding that Hispanic participants were significantly more likely than non-Hispanic white participants to report several ACEs categories, including parental divorce/separation, physical and sexual abuse, parental incarceration, and physical and emotional neglect [29]. Mersky and colleagues [29] found that white and Asian participants did not differ in mean ACEs scores, in contrast to the current finding that Asian patients had lower ACEs scores than white patients, although other studies have found that Asian/Pacific Islander college students report higher numbers of certain ACEs than white students [30]. However, the number of Asian and Hispanic patients in the current study was quite low compared to the number of white patients, possibly tempering the conclusions that can be drawn from these findings. Consistent with most previous research, patients with BED reported higher total ACEs scores than patients with AN-R.

LCA allowed us to examine clusters of ACEs unique to different individual patients, rather than examining correlations across the groups as a whole. LCA revealed four profiles, with the largest subgroup belonging to the “Low ACEs” profile. However, over half of the sample fell into one of the other three groups characterized by higher levels of ACEs. Using this method allowed us to see that, whereas some patients may be categorized simply by having “few” or “many” ACEs, other patients may be better classified by endorsement patterns indicative of direct abuse or household stressors. Given that only some patients endorsed these patterns, the patterns themselves would be unlikely to emerge with more variable-centered analyses that restrict patterns to be observable across the full sample.

Compared to patients with AN-R, patients with OSFED were more likely to fall into the profile characterized by high levels of household dysfunction. A diagnosis of OSFED is given when patients have clinically significant problems with eating or weight, but do not meet criteria for AN, BN, or BED. Examples of OSFED include “atypical AN”, in which a person meets criteria for AN but his or her weight is in a normal range, or subclinical BN, in which a person does not meet criteria for the frequency and/or duration of binge eating/purging behaviors. Little research has been done on ACEs specifically in patients with OSFED, although OSFED and co-occurring posttraumatic stress disorder (PTSD) have been found to be associated with premature treatment dropout from a day hospital program [31]. This suggests that trauma among patients with OSFED has clinical implications and should be carefully assessed at the onset of treatment. Trauma related to family dysfunction may be particularly relevant for this population. However, the nature of the OSFED diagnosis means that patients with this diagnosis may have heterogeneous presentations and a variety of clinical characteristics. We were unable to classify this subgroup further in the current study. Future research is needed to replicate these findings and examine results more closely according to the subtype of OSFED, e.g., atypical AN, subclinical BN, subclinical BED, etc.

Compared to patients with AN-R, those with BED were more likely to fall into the profile characterized by high levels of emotional and physical ACEs. Previous studies have found high levels of lifetime traumatic experiences among patients with BED (73.2–91.5%) [32–35] and have found associations between a history of trauma and poor treatment outcome for this population [35, 36]. Further research is needed to determine the nature of the association between BED and high levels of trauma, and also between specific types of trauma and specific types of EDs. For example, it is unclear why BED in particular would be associated with high levels of psychological and physical ACEs as opposed to other types of ACEs. The four LCA profiles also raise questions about why certain types of ACEs tend to co-occur, and how early interventions might target several ACEs at once.

Limitations of the current study include self-report and retrospective assessment of ACEs. Although this study is one of the first to use the Adverse Childhood Experiences Survey [3] in an ED sample, the survey does not address forms of trauma such as childhood bullying, which can also have long-term associations with poor mental health [37]. Meta-analyses have found moderate effect sizes for the relationship between weight-related teasing and ED behaviors [38], and have found that individuals with EDs were significantly more likely than healthy controls to have been bullied or teased prior to the onset of their ED [39]. In addition, the Adverse Childhood Experiences Survey does not assess trauma occurring after the age of 18. The mean age of the current sample was 27; it is possible that EDs developed after the occurrence of adulthood trauma. The Adverse Childhood Experiences Survey also does not assess the degree to which the respondent was traumatized by the event or whether the respondent chose to confide in someone about the trauma, as does the Childhood Trauma Questionnaire [40]. Also, we assessed patients from two private ED treatment facilities, possibly limiting the generalizability of our findings. Although all patients who received treatment and signed consent were included in the current study, patients need to either use insurance or pay out-of-pocket for treatment, which may have impacted the representativeness of the patient sample. In addition, there was a high proportion of patients with AN in the sample, which may be expected in a treatment program offering higher levels of care, but is not representative of EDs in the population at large [27]. Additionally, due to the number of different tests performed, several different corrections for multiple comparisons could have also been performed and would have rendered a few of the findings statistically nonsignificant. Finally, we were unable to examine the association between history of ACEs and treatment outcome, level of care, or the association between body mass index (BMI) and ACEs, as previous studies have found that childhood trauma is positively associated with greater BMI [41]. These are important areas for future research. Future research should also replicate findings from the current study and investigate possible reasons behind the often-lower rates of ACEs found among participants with AN-R compared to other EDs to identify possible protective or resilience factors. Strengths of the study include the large sample size and the representation of all ED diagnoses, including OSFED and ARFID, which have historically been understudied in the context of examining ACEs. Identification of profiles by LCA is an additional strength of the study, as it allows for examination of more complex patterns of ACEs rather than examining specific types of abuse, such as emotional or physical, separately.

## Conclusions

The current study supports the assertion that there are high rates of ACEs among patients with EDs, and adds findings that certain ED diagnoses, such as BED, may be associated with specific types of trauma. Screening for ACEs at admission into an ED program could shape the focus of individual treatment or direct the patient into appropriate trauma-focused group therapy. This study is one of the first to examine ACEs using the Adverse Childhood Experiences Survey in a large transdiagnostic sample of individuals with EDs. There is evidence that childhood trauma may be associated with different consequences than adult trauma [42], suggesting that examining childhood trauma specifically, as is assessed by the Adverse Childhood Experiences Survey, may provide important insights that might not be captured by examining trauma across the lifespan or solely in adulthood.

Prevention of ACEs is a significant public health challenge. Providing children with safe household environments may lessen the long-term development of a number of serious disorders, including EDs. Primary prevention efforts, starting in childhood, are key to the vital endeavor of improving the well-being of children well into adulthood.

## Supplementary Information

**Additional file1: Table S1** ED Female Sample Compared to Nationally Representative Female Sample. **Table S2** ED White Sample Compared to Nationally Representative White Sample. **Table S3** Latent Class Solution Fit Indices.

## Data Availability

The dataset used and analyzed for the current study is available from the corresponding author upon reasonable request.

## Abbreviations

ACE: Adverse childhood experience
ACES: Adverse childhood experiences survey
AIC: Akaike's information criterion
AN: Anorexia nervosa
AN-BP: Anorexia nervosa—binge/purge subtype
AN-R: Anorexia nervosa—restricting subtype
ARFID: Avoidant/restrictive food intake disorder
BED: Binge eating disorder
BIC: Bayesian information criterion
BMI: Body mass index
BN: Bulimia nervosa
CI: Confidence interval
DSM-5: Diagnostic and statistical manual of mental disorders—5th edition
ED: Eating disorder
ERC: Eating recovery center
LCA: Latent class analysis
OR: Odds ratio
OSFED: Other specified feeding or eating disorder
PTSD: Posttraumatic stress disorder
TIC: Trauma-informed care
