# Correction: Adverse childhood experiences among adults with eating disorders: comparison to a nationally representative sample and identification of trauma profiles

**DOI:** 10.1186/s40337-022-00639-1

**Published:** 2022-08-08

**Authors:** Renee D. Rienecke, Craig Johnson, Daniel Le Grange, Jamie Manwaring, Philip S. Mehler, Alan Duffy, Susan McClanahan, Dan V. Blalock

**Affiliations:** 1 Eating Recovery Center, Pathlight Mood & Anxiety Centers, Denver, USA; 2 Department of Psychiatry and Behavioral Sciences, Northwestern University, 333 N. Michigan Avenue, Ste. 1900, Denver, IL 60601 USA; 3 Department of Psychiatry and Behavioral Sciences, University of California, San Francisco, CA USA; 4 Department of Psychiatry and Behavioral Neuroscience, The University of Chicago, Chicago, IL (Emeritus) USA; 5 ACUTE, at Denver Health, Denver, CO USA; 6 Department of Medicine, University of Colorado, Denver, CO USA; 7 Rush University Medical Center, Denver, IL USA; 8 Center of Innovation to Accelerate Discovery and Practice Transformation, Durham Veterans Affairs Medical Center, Durham, NC USA; 9 Department of Psychiatry and Behavioral Sciences, Duke University School of Medicine, Durham, NC USA

## Correction to: Journal of Eating Disorders (2022) 10:72 https://doi.org/10.1186/s40337-022-00594-x

Following the publication of the original article [1], the author reported that the word 'profiles' was omitted from the article title.

The correct title should read: "Adverse childhood experiences among adults with eating disorders: comparison to a nationally representative sample and identification of trauma profiles".

The correct title has been captured both in this Correction and the original article, which has now been revised.
