# Supplementary material for: Adverse childhood experiences among adults with eating disorders: comparison to a nationally representative sample and identification of trauma
Source: J Eat Disord. 2022 May 20;10:72. doi: 10.1186/s40337-022-00594-x (PMC9123748; doi:10.1186/s40337-022-00594-x)
Supplement: Supplementary file 1 — Additional file1: Table S1 ED Female Sample Compared to Nationally Representative Female Sample. Table S2 ED White Sample Compared to Nationally Representative White Sample. Table S3 Latent Class Solution Fit Indices. [file 40337_2022_594_MOESM1_ESM.docx]

Supplementary Tables

| Supplementary Table 1. *ED Female Sample Compared to Nationally Representative Female Sample* | | | | | | | | | |
| --- | --- | --- | --- | --- | --- | --- | --- | --- | --- |
| Sample | **ACEs M (SD)** | Emo Abuse | Phys Abuse | **Sex Abuse** | **Divorce** | **IPV** | Sub Use | **Mental Illness** | **Prison** |
| ED F | **1.98 (1.92)** | 35% | 17% | **20%** | **36%** | **9%** | 30% | **42%** | 6% |
| Nat’l F | **1.68 (4.54)** | 33% | 17% | **16%** | **27%** | **18%** | 28% | **19%** | 7% |
| Endorsement in ED Total Sample Compared to Nationally Representative Total Sample | | | | | | | | | |
| Test | t-test,  p-value | OR, 95% CI | OR, 95% CI | OR, 95% CI | OR, 95% CI | OR, 95% CI | OR, 95% CI | OR, 95% CI | OR, 95% CI |
| Result | **t=4.68, p<.00001**  **cohen’s d=.07** | 1.08 [0.94, 1.12] | 1.01 [0.85, 1.19] | **1.31 [1.12, 1.54]** | **1.46 [1.28, 1.68]** | **0.46 [0.37, 0.57]** | 1.09 [0.94, 1.25] | **3.12 [2.73, 3.55]** | 0.85 [0.65, 1.11] |
| *Note.* Bolded numbers represent significant differences. “ED F” refers to only the female patients in the sample of patients seeking treatment for ED. “Nat’l F” refers to only the female participants in the nationally representative sample described in Merrick et al., 2018 [1]. “Divorce” refers to household divorce. “IPV” refers to a mother or stepmother in the household experiencing intimate partner violence. “Sub Use” refers to having a family member in the household engaging in illicit drug use or struggling with alcoholism. “Mental Illness” refers to having a family member in the household who struggles with mental illness or has attempted suicide. “Prison” refers to having a family member in the household who is incarcerated. | | | | | | | | | |

| Supplementary Table 2. *ED White Sample Compared to Nationally Representative White Sample* | | | | | | | | | |
| --- | --- | --- | --- | --- | --- | --- | --- | --- | --- |
| Sample | **ACEs M (SD)** | Emo Abuse | Phys Abuse | **Sex Abuse** | **Divorce** | **IPV** | Sub Use | **Mental Illness** | **Prison** |
| ED W | **1.96 (1.93)** | 35% | 17% | **19%** | **36%** | **9%** | 29% | **42%** | 5% |
| Nat’l W | **1.52 (4.32)** | 33% | 17% | **16%** | **27%** | **18%** | 28% | **19%** | 7% |
| Endorsement in ED Total Sample Compared to Nationally Representative Total Sample | | | | | | | | | |
| Test | t-test,  p-value | OR, 95% CI | OR, 95% CI | OR, 95% CI | OR, 95% CI | OR, 95% CI | OR, 95% CI | OR, 95% CI | OR, 95% CI |
| Result | **t=6.69, p<.000000001**  **cohen’s d=.10** | 1.03 [0.90, 1.18] | 1.03 [0.87, 1.23] | **1.88 [1.59, 2.22]** | **1.66 [1.45, 1.90]** | **0.57 [0.46, 0.71]** | 1.08 [0.94, 1.25] | **3.23 [2.82, 3.69]** | 0.85 [0.64, 1.14] |
| *Note.* Bolded numbers represent significant differences. “ED W” refers to only the white patients in the sample of patients seeking treatment for ED. “Nat’l W” refers to only the white participants in the nationally representative sample described in Merrick et al., 2018 [1]. “Divorce” refers to household divorce. “IPV” refers to a mother or stepmother in the household experiencing intimate partner violence. “Sub Use” refers to having a family member in the household engaging in illicit drug use or struggling with alcoholism. “Mental Illness” refers to having a family member in the household who struggles with mental illness or has attempted suicide. “Prison” refers to having a family member in the household who is incarcerated. | | | | | | | | | |

| Supplementary Table 3. *Latent Class Solution Fit Indices* | | | | | | |
| --- | --- | --- | --- | --- | --- | --- |
| # of Classes | -2 LL | Residual DF | AIC | BIC | χ^2^ | Entropy |
| 1 | -4330.31 | 247 | 8676.61 | 8716.61 | 26355.80 | -- |
| 2 | -3871.68 | 238 | 7777.37 | 7861.81 | 507.04 | .74 |
| 3 | -3819.14 | 229 | 7690.28 | 7819.42 | 373.98 | .61 |
| **4** | **-3784.69** | **220** | **7639.37** | **7813.21** | **262.77** | **.67** |
| 5 | -3774.33 | 211 | 7636.66 | 7855.21 | 221.13 | .63 |
| 6 | -3766.20 | 202 | 7636.66 | 7901.66 | 202.24 | .73 |
| 7 | -3758.57 | 193 | 7638.41 | 7949.09 | 202.00 | .60 |
| *Note.* Bolded row represents chosen Latent Class solution. | | | | | | |
